# Generation of peanut mutants by fast neutron irradiation combined with *in vitro* culture

**DOI:** 10.1093/jrr/rru121

**Published:** 2015-02-04

**Authors:** Jing-Shan Wang, Jiong-Ming Sui, Yong-Dun Xie, Hui-Jun Guo, Li-Xian Qiao, Li-Lan Zhao, Shan-Lin Yu, Lu-Xiang Liu

**Affiliations:** 1 College of Life Sciences, Qingdao Agricultural University/Key Lab of Plant Biotechnology in Universities of Shandong Province, Qingdao 266109, China; 2 Institute of Crop Science, Chinese Academy of Agricultural Sciences/National Key Facility for Crop Gene Resources and Genetic Improvement, National Center of Space Mutagenesis for Crop Improvement, Beijing 100081, China; 3 Shandong Agricultural Broadcasting and Television School, Penglai 265600, China; 4 Peanut Research Institute, Shandong Academy of Agricultural Sciences, Qingdao 266100, China

**Keywords:** peanut (*Arachis hypogaea* L.), fast neutron irradiation, mutagenesis, *in vitro* culture, mutant

## Abstract

Induced mutations have played an important role in the development of new plant varieties. In this study, we investigated the effects of fast neutron irradiation on somatic embryogenesis combined with plant regeneration in embryonic leaflet culture to develop new peanut (*Arachis hypogaea* L.) germplasm for breeding. The dry seeds of the elite cultivar Luhua 11 were irradiated with fast neutrons at dosages of 9.7, 14.0 and 18.0 Gy. The embryonic leaflets were separated and incubated in a medium with 10.0-mg/l 2,4-D to induce somatic embryogenesis. Next, they were incubated in a medium with 4.0-mg/l BAP for plant regeneration. As the irradiation dosage increased, the frequency of both somatic embryo formation and plantlet regeneration decreased. The regenerated plantlets were grafted onto rootstocks and were transplanted into the field. Later, the mature seeds of the regenerated plants were harvested. The M_2_ generation plants from most of the regenerated cultivars exhibited variations and segregation in vigor, plant height, branch and pod number, pod size, and pod shape. To determine whether the phenotypes were associated with genomic modification, we compared the DNA polymorphisms between the wild-type plants and 19 M_3_-generation individuals from different regenerated plants. We used 20 pairs of simple sequence repeat (SSR) primers and detected polymorphisms between most of the mutants and the wild-type plants (Luhua 11). Our results indicate that using a combination of fast neutron irradiation and tissue culture is an effective approach for creating new peanut germplasm.

## INTRODUCTION

Induced mutations have been an important method for creating new germplasm resources and accelerating crop breeding [1, 2]. Radiation mutagenesis can promote gene recombination, break undesirable gene linkages, and produce DNA mutations [3, 4] or chromosome variations [5]. Using a combination of radiation mutagenesis with *in vitro* culture can increase the genetic diversity of the breeding population and reduce chimerism [2, 6–9]. With the development of biotechnology, *in vitro* mutagenesis has become increasingly valuable for breeding, and radiation mutagenesis in combination with *in vitro* culture has been used to create elite crop varieties [6, 10]. This study uses a combination of radiation mutagenesis with *in vitro* culture to produce new peanut germplasm for breeding.

Peanut (*Arachis hypogaea* L.) is an important oil crop. Increasing land salinization and drought have focused breeding objectives on the development of high-yielding, stress-tolerant peanut cultivars. However, obtaining high-yielding, stress-tolerant peanut cultivars with conventional breeding methods is difficult, as stress-resistant germplasm is scarce due to the limited genetic diversity in the peanut [11].

Fast neutron irradiation, a form of ionizing radiation with a high linear energy density, can cause secondary ionization and gene mutations in plant cells, and the traits of the resulting mutants can be stably inherited [12–14]. In this study, we examined the effects of a combination of fast neutron irradiation and *in vitro* culture on peanut somatic embryogenesis and plant regeneration. We also analyzed the segregation and variation in the offspring (M_2_ generation) of the regenerated plantlets.

## MATERIALS AND METHODS

### Peanut cultivars, tissue culture media, and conditions

The seeds of the Luhua 11 peanut cultivar were obtained from an experimental field at Qingdao Agricultural University, in Qingdao, China. The somatic embryo induction medium contained MS basal salts, B_5_ vitamins, 3% sucrose, 0.8% agar, and 10 mg/l 2,4-dichlorophenoxyacetic (2, 4-D). The germination medium contained MS basal salts, B_5_ vitamins, 3% sucrose, 0.8% agar, and 4 mg/l 6-benzyl amino purine (BAP). The pH of all media was 5.8. All of the *in vitro* cultures were maintained in a growth room stabilized at 25 ± 1°C with a photoperiod of 13 h light.

### Fast neutron irradiation, *in vitro* cultures, and plant regeneration

Neutrons were produced from the reaction of T(d, n)_α_, with a neutron energy of 14 MeV. The irradiation dose was calculated by monitoring the accompanying a particles [15]. The mature, plump, and air-dried seeds were subjected to 0, 9.7, 14.0 and 18.0 Gy of fast neutron irradiation. The cotyledons were removed from the seeds, and the embryos were surface-disinfected then soaked in sterile water for 12–16 h before dissection. The embryonic leaflets (explants) were dissected from the embryos and cultured in a somatic embryo induction medium. There were 32 explants for each treatment. Four weeks later, the surviving explants, with and without somatic embryos, were transferred to the somatic embryo germination medium. The somatic embryo induction rates and the plantlet regeneration rates were determined 4 weeks and 8 weeks later using the following equations: the somatic embryo induction rate (%) = the number of explants forming somatic embryos/the number of explants × 100%; and the plantlet regeneration rate (%) = the number of explants regenerating plantlets/the number of explants × 100%.

In these equations, the number of explants refers to the initial number of embryonic leaflets placed in the induction medium.

### Grafting and transplanting the regenerated plantlets

When the regenerated plantlets grew to a height of 2 cm, we resected them from the base and used them as scions. The rootstocks were taken from 10- to 12-day-old peanut seedlings of Huayu 23. The grafted plantlets were cultured under sterile conditions for 1–2 days and were subsequently transferred to pots containing a mixture of soil:vermiculite:turf (1:1:1) for acclimation in a greenhouse at 24 ± 2°C [16].

After growing in the greenhouse for 3 weeks, the grafted plants were transplanted into an experimental field at Qingdao Agricultural University. All of the seeds (M_2_ generation) produced by the regenerated plants were harvested and sown in the field the next year. In total, 826 M_2_ seeds were sown. Among them, 429, 265 and 132 seeds were obtained from 9.7-Gy, 14.0-Gy and 18.0-Gy–irradiated plants respectively. The phenotypes of the individual M_2_ plants were recorded throughout the growing season.

### DNA extraction, purification, and PCR amplification

The young leaves of 3-week-old M_3_ plants (one plant from each of the regenerated plants and Luhua 11) were sampled and immediately placed in liquid nitrogen. The DNA was isolated using the CTAB method, as previously described [17]. The DNA concentration was adjusted to 50 ng/µl of PCR amplification. DNA polymorphism was detected using 20 previously described [18] primer pairs.

For the simple sequence repeat (SSR) analysis, the reaction mixture (10 μl) contained 1.0 μl of 10 × PCR buffer (2 mM of MgCl_2_), 10 ng of genomic DNA, 200 μM of deoxynucleotide triphosphates (dNTPs), 0.3 μM of primers, and 0.1 units of Taq polymerase. The samples were subjected to the following thermal profile for amplification: initial denaturing at 94°C for 5 min, followed by 35 cycles of 94°C for 1 min, 55°C for 45 s, and 72°C for 1 min; then, a final elongation was conducted at 72°C for 5 min.

When the PCR cycles were complete, 5.0 μl of loading dye (containing 98% deionized formamide, 10 mM EDTA, 0.25% bromophenol blue, and 0.25% xylene cyanol) was added to the reaction mixture. A 3-μl aliquot was loaded into a 6% polyacrylamide sequencing gel. Electrophoresis was performed at 80 W for 2 h. The gel was visualized with silver staining [19].

## RESULTS

### Effects of fast neutron irradiation on somatic embryogenesis and plant regeneration

**Fig. 1.**
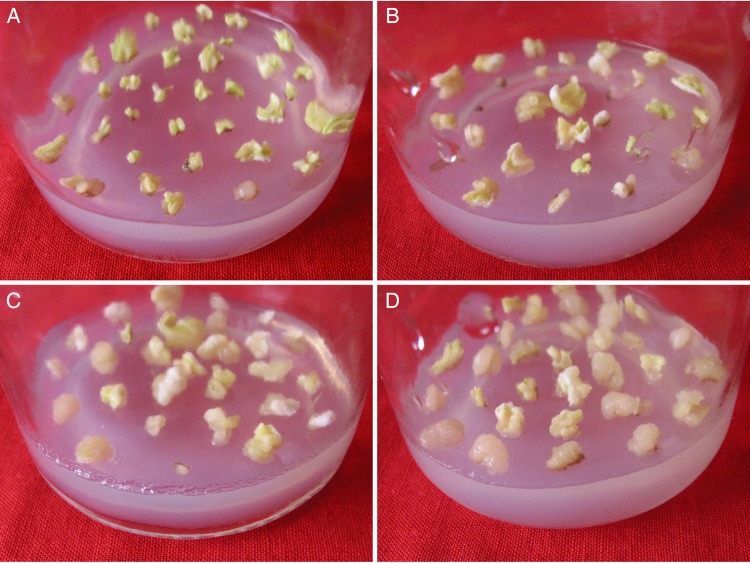
Formation of somatic embryos and calli. Explants (leaflets) were obtained from seeds exposed to one of four doses of fast neutron irradiation: (**A**) 0 Gy, (**B**) 9.7 Gy, (**C**) 14.0 Gy and (**D**) 18.0 Gy. Representative explants were photographed after 4 weeks on the somatic embryo induction medium.

The explants (embryonic leaflets) obtained from the irradiated seeds of Luhua 11 were cultured on the somatic embryo induction medium. They began to form loose calli by the seventh day, and somatic embryos were formed by the 15th day. As the radiation dosage increased, the number of explants that formed somatic embryos decreased, but the number that formed calli increased (Fig. 1).

**Fig. 2.**
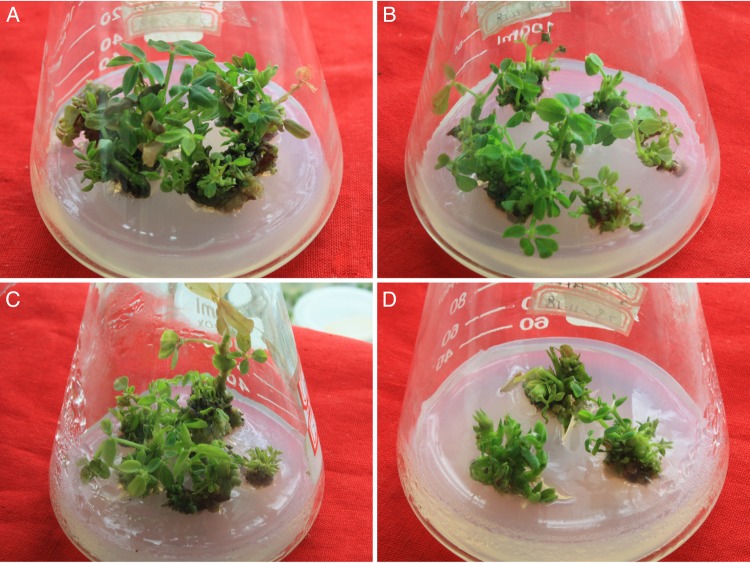
Representative plantlets developed from somatic embryos. The original Luhua 11 seeds were subjected to fast neutron irradiation at (**A**) 0 Gy, (**B**) 9.7 Gy, (**C**) 14.0 Gy and (**D**) 18.0 Gy.

After being cultured in the somatic embryo induction medium for 4 weeks, the explants with somatic embryos or calli were transferred to the somatic embryo germination medium. The somatic embryos gradually developed into mature embryos, germinated, and developed into plantlets. After being transferred to the germination medium, some calli that did not previously produce somatic embryos formed somatic embryos, germinated, and developed into plantlets (Fig. 2). As indicated in Table 1, the somatic embryo induction rates decreased with increasing radiation dosage. The plantlet regeneration frequency, which was determined 8 weeks after transferring to germination medium, also declined as the radiation dosage increased (Table 1).

**Table 1.** The effects of fast neutron irradiation on somatic embryo induction and plant regeneration in peanut cultivar Luhua 11

Irradiation dosage (Gy)	Somatic embryo induction rate (%)	Plantlet regeneration rate (%)
0	92.3	79.8
9.7	56.7	31.7
14.0	40.0	24.6
18.0	33.3	19.8

### Grafting of regenerated plantlets and their growth in the field

**Fig. 3.**
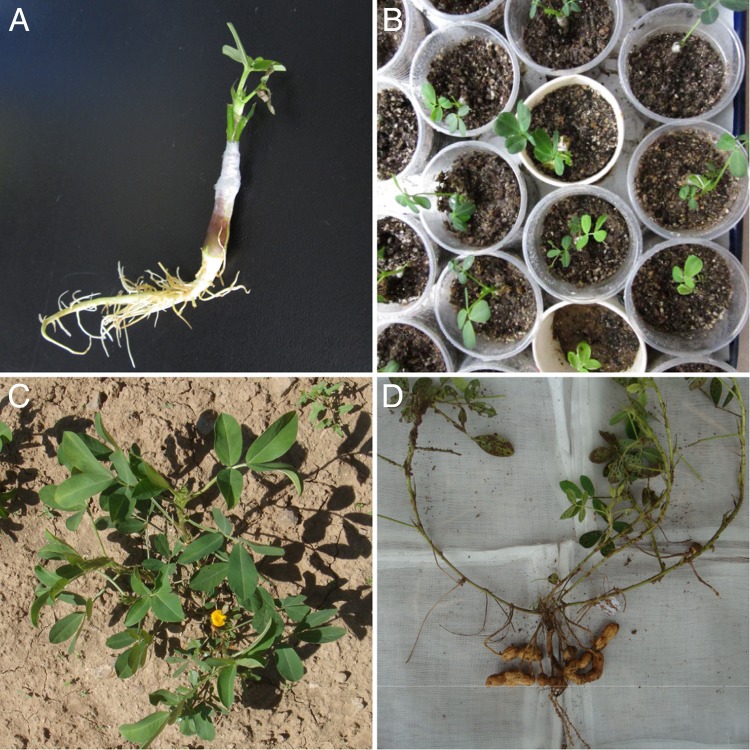
Grafting of regenerated plantlets and their growth in the field. (**A**) A grafted plantlet (the scion was from a somatic embryo of Luhua 11 and the rootstock was from the Huayu 23 cultivar). (**B**) Acclimation of grafted, regenerated plantlets in the greenhouse. (**C**) A regenerated plantlet (M_1_ generation) growing in the field. (**D**) A regenerated M_1_ plant that produced pods.

When the regenerated plantlets grew to 1–2 cm, the plantlets were aseptically grafted onto the rootstocks of Huayu 23 as scions (Fig. 3A). After acclimation in a greenhouse for 3 weeks (Fig. 3B), the grafted plantlets were transplanted into the field (Fig. 3C). The mature pods of the regenerated plantlets (the M_1_ generation) were harvested from each plant (Fig. 3D). A total of 125 grafted plantlets produced pods; 49 plantlets (No. 57–105) came from explants exposed to 9.7 Gy, 35 plantlets (No. 1–35) came from explants exposed to 14.0 Gy, 21 plantlets (No. 36–56) came from explants exposed to 18.0 Gy, and 20 plantlets came from non-irradiated explants.

### Segregation and variation of M_2_ plants

The M_2_ plants were monitored for growth, development and agronomic traits. In this study, a total of 655 M_2_ plants were analyzed. Of these, 345 plants came from the 9.7-Gy dose, 209 plants came from the 14.0-Gy dose, and 101 plants came from the 18.0-Gy dose. Most of the offspring of the original regenerated plants displayed significant phenotypes in terms of seedling vigor and plant height, as well as pod size, shape, weight, and pod number of each plant.

**Fig. 4.**
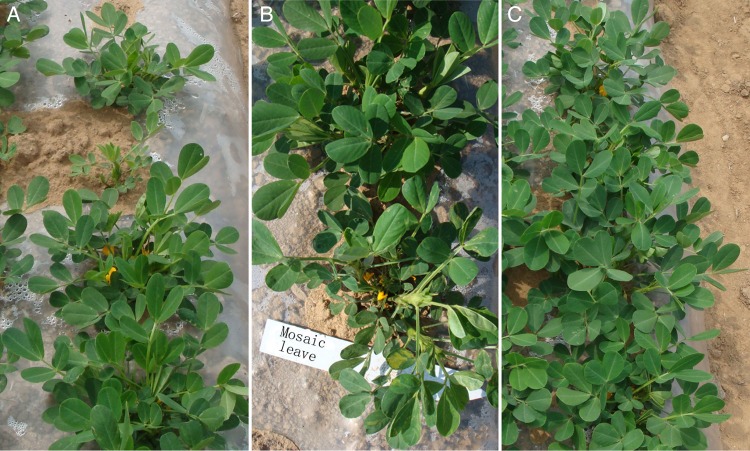
Trait variation among the M_2_ plants at the seedling stage. (**A**) The offspring of Plant No. 58 (derived from an explant exposed to 9.7 Gy of fast neutron irradiation) exhibited segregation in growth vigor and flowering date. (**B**) One offspring of Plant No. 7 (derived from an explant exposed to 14.0 Gy of fast neutron irradiation) produced leaves with a mosaic pattern, whereas other offspring from the same plant produced normal leaves. (**C**) The wild-type Luhua 11 plants produced offspring that were uniform in growth and appearance.

Segregation among the M_2_ plants was evident. For example, the M_2_ plants from the 9.7-Gy dose derived from the M_1_ Plant No. 58 differed in seedling vigor, growth rate, and flowering time (Fig. 4A). At the 14.0-Gy dose, some of the M_2_ plants produced from M_1_ Plant No. 7 produced large leaves or leaves with a mosaic pattern (Fig. 4B). In contrast, wild-type Luhua 11 seeds produced plants with uniform growth and appearance (Fig. 4C). A comparison of the wild-type and non-irradiated regeneration plants did not identify any trait variations (data not shown). Therefore, the wild-type plants were used as a control for the following experiments.

**Fig. 5.**
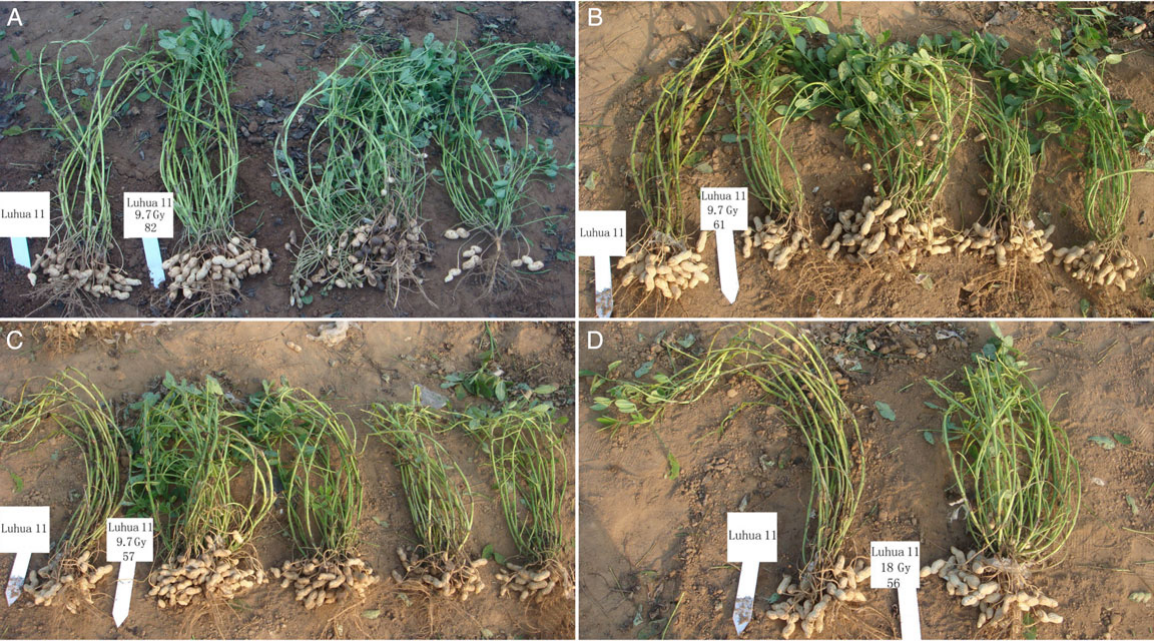
Variation in plant height, plant architecture and branch number in the M_2_ plants at harvest. (**A**) The offspring of Plant No. 82 (derived from an explant exposed to 9.7 Gy of fast neutron irradiation) had closely spaced branches. (**B**) The offspring of Plant No. 61 showed segregation in branch number (derived from an explant exposed to 9.7 Gy of fast neutron irradiation). (**C**) The offspring of Plant No. 57 were short and showed segregation in branch number (derived from an explant exposed to 9.7 Gy of fast neutron irradiation). (**D**) One offspring of Plant No. 56 was short with closely spaced branches (derived from an explant exposed to 18.0 Gy of fast neutron irradiation). The wild-type Luhua 11 plants are on the left in each photograph.

We characterized the traits from the M_2_ plants at late stages of development and at harvest. If the trait mean of a certain M_2_ plant deviated from the range of control plants by 15%, we defined them as mutants. As shown in Table 2, the mutant frequency of the shoot traits was 7.8% for main stem height, 4.7% for branch length, 3.4% for total branch number, and 7.6% for yielding branch number. Both plant height and branch number differed in some offspring from the same regenerated plant. For example, at the 9.7-Gy dose, some offspring of Plant No. 82 had closely spaced branches (Fig. 5A), whereas some offspring of Plant No. 57 and Plant No. 61 were short and had more branches than the wild-type control. Some offspring had 19 and 26 branches, whereas other offspring from the same plant had 7 to 9 branches per plant (Fig. 5B and C). At the 18.0-Gy dose, one offspring of Plant No. 56 was short and had closely spaced branches (with 22 branches in total) (Fig. 5D). The wild-type plant, Luhua 11, had an average of 12 branches per plant.

**Table 2.** Mutant frequency of agronomic traits in the M_2_ population of Luhua 11

Item	Stem height	Branch length	Total branch number	Yielding branch number	Pod number per plant	Single-plant yield	Hundred-pod weight	Pod shape
Number of mutants	51	31	22	50	79	70	74	98
Mutant frequency (%)	7.8	4.7	3.4	7.6	12.0	10.7	11.3	14.9

A total of 655 M_2_ plants were screened. Mutant frequency = number of mutants identified/the number of total M_2_ plants screened × 100%.

**Fig. 6.**
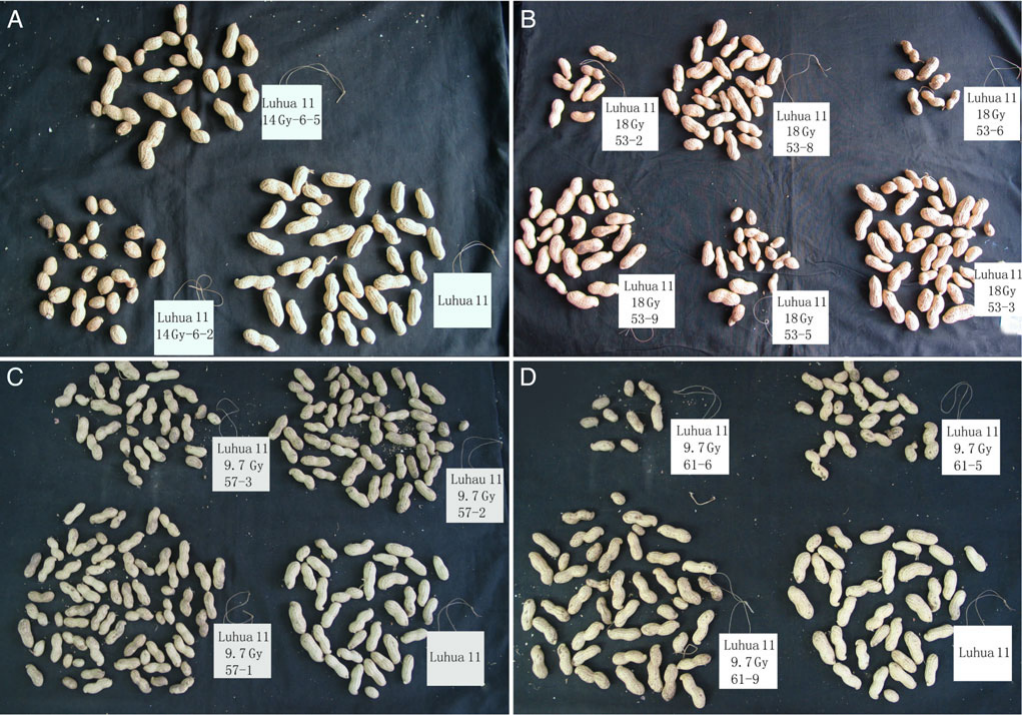
Pods produced by single M_2_ plants and the wild-type plants, Luhua 11. (**A**) Pods produced by the offspring of Plant No. 6 had very narrow ‘waists’ or were gourd-shaped (derived from an explant exposed to 14.0 Gy of fast neutron irradiation). (**B**) Pods produced by the offspring of Plant No. 53 showed variation and segregation in pod shape and size (derived from an explant exposed to 18.0 Gy of fast neutron irradiation). (**C**) Offspring 57-1 and 57-2 of Plant No. 57 produced more pods than Luhua 11 and the other offspring (derived from an explant exposed to 9.7 Gy of fast neutron irradiation). (**D**) Pods produced by the offspring of Plant No. 61 showed variation and segregation in shape and size. In addition, 61-9 produced more pods than Luhua 11 and the other offspring (derived from an explant exposed to 9.7 Gy of fast neutron irradiation). Note, the mutants are designated by two numbers (separated by a dash). The first number refers to the regenerated plant first transplanted in the field, and the second number refers to a specific offspring of that plant.

Compared with the shoot traits of the M_2_ plants, a higher mutant frequency for the traits of underground parts was observed (listed in Table 2). The mutant frequency was 12% for pod number per plant, 10.7% for single-plant yield, 11.3% for hundred pod weight, and 14.9% for pod shape (such as gourd, cocoon, narrow waist, or axe shape). Pod number, weight and shape differed between the offspring of the regenerated plants and Luhua 11. For example, some offspring of Plant No. 6 produced gourd-shaped pods, whereas others produced pods with extremely narrow ‘waists’ (Fig. 6A). The offspring of Plant No. 53 exhibited variation and segregation in both pod shape and size (Fig. 6B). The offspring of Plant No. 57 produced different numbers of pods. Two plants, 57-1 and 57-2, produced more pods than the other offspring and wild-type plants (Fig. 6C). One offspring (61-9) of Plant No. 61 produced more pods than the other offspring of the same plant or wild-type plants (Fig. 6D).

### Single-plant yield and pod weight trait inheritance in the M_3_ plants

We measured the single-plant yield of M_3_ plants generated from high-yielding M_2_ plants (1-5, 46-1, 46-2, 46-3, 57-1 and 57-2), all of which produced >50 g per plant. The results are listed in Table 3. Although this trait showed segregation in the M_3_ generation, some plants had high single-plant yield. The hundred pod weight was also analyzed in this study. The hundred pod weight of the M_2_ plant 42-1 was 126 g, and the average weight of its M_3_ offspring was 128 g. In the M_2_ generation, both 16-4 and 16-5 produced large pods with a hundred pod weight of 309 g and 286 g. The hundred pod weight of their M_3_ generation was 307 g and 294 g. These results indicated that both single-plant yield and pod weight traits could be inherited.

**Table 3.** Transmission of single-plant yield in the M_3_ generation

M_2_ individual codes	Number of M_3_ plants that produced indicated single-plant yield			
	35.0–40.0 g	40.1–50.0 g	50.1–60.0 g	>60.0 g
1-5	0	2	2	1
46-1	1	1	2	0
46-2	2	2	1	1
46-4	0	0	1	2
57-1	0	6	5	3
57-2	1	3	2	1

The single-plant yield of M_2_ plants listed in this table was >50.0 g, and the average single-plant yield of control plants (Luhua 11) was 35.7 g.

### SSR analyses between the wild-type plants and mutants

The SSR analyses were performed to compare the wild-type plants (Luhua 11) with the 19 M_3_ generation individuals derived from 19 regenerated plants using 20 primer pairs. Nine SSR markers displayed polymorphisms between mutants and wild-type plants (Table 4). The SSR marker polymorphisms represented the following changes: (i) a decrease in the number of amplified fragments, (ii) an increase in the number of fragments, and (iii) a change in fragment size with a frequency of 47.5%, 14.7% and 37.8%, respectively. The polymorphism rate induced by irradiation at 14.0 Gy was higher than at either 9.7 Gy or 18.0 Gy.

**Table 4.** SSR polymorphic results between the selected mutants and control plants (Luhua 11)

SSR markers	CK	57-4	65-1	72-4	84-3	90-2	98-4	1-3	6-4	14-5	23-5	32-2	35-2	37-4	41-1	44-5	48-3	51-5	53-1	56-2
PM377	–	–	–	–	–	–	–	C	–	–	–	C	A	–	–	A	–	–	C	–
PM297	–	–	–	–	–	–	C	A	A	B	C	C	A	A	–	–	–	–	A	–
PM348	–	–	–	C	–	C	–	A	A	C	C	A	A	A	–	C	–	–	C	–
WH091	–	–	–	–	–	–	–	C	C	–	–	–	–	C	–	–	–	–	C	–
WH070	–	–	–	–	A	A	A	–	–	A	A	–	–	–	A	A	A	A	–	A
PM660	–	–	–	–	–	A	–	–	–	–	–	–	–	–	–	A	C	C	C	C
WH039	–	–	–	–	–	–	–	–	–	–	–	C	C	–	–	–	–	–	–	–
WH015	–	–	–	–	B	B	–	–	–	–	–	–	–	–	–	–	–	–	–	C
WH090	–	–	–	–	–	–	B	B	B	B	B	B	A	A	A	A	A	–	–	–

CK = control plants, A = a decrease in the number of amplified fragments, B = an increase in the number of amplified fragments, C = a size change of the amplified fragments.

The samples of 57-4, 65-1, 72-4, 84-3, 90-2 and 98-4 were from the 9.7 Gy radiation dose; the samples of 1-3, 6-4, 14-5, 23-5, 32-2 and 35-2 were from the 14.0 Gy radiation dose; and the samples of 37-4, 41-1, 44-5, 48-3, 51-5, 53-1 and 56-2 were from the 18.0 Gy radiation dose.

**Fig. 7.**
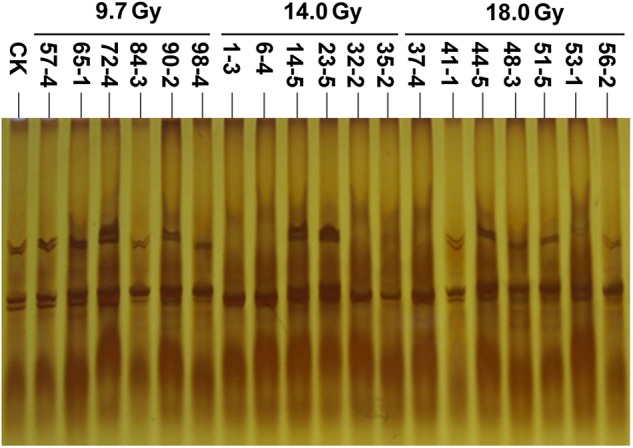
Amplification patterns of SSR marker PM348 for mutants and the wild-type Luhua 11 plants (CK).

The ability to use the SSR markers to distinguish wild-type plants from mutants was evaluated. WH039 and WH015 identified the variation in two and three mutants (Table 4). The other markers revealed variations in more than four mutants. PM297, for example, revealed variations in nine mutants. Both WH090 and PM348 revealed variations in 11 mutants (Table 4 and Fig. 7).

## DISCUSSION

For the creation and screening of new germplasm resources, a combination of induced mutagenesis and tissue culture can save labor and other resources [20, 21]. This combination can also reduce chimerism, and the resulting mutant plants can be directly used to breed new crop varieties [22–25]. The embryonic calli from wheat rataria exposed to gamma radiation has been screened; the resulting embryos were grown in a tissue culture to obtain regenerated plants that were high yielding, early maturing, disease resistant, and waterlogging tolerant [22]. Using mutagenesis in combination with tissue culture, ‘Xiaguang’ and 13 other new elite varieties of chrysanthemum were successfully developed [26]. Pan cultivated two small and dwarf mutants of the Chinese orchid using different radiation dosages and tissue culture [24]. In previous studies, we described the effect of the mutagen pingyangmycin (PYM) on somatic embryo formation in peanut tissue culture and determined the optimal screening concentration of hydroxyproline (HYP) for obtaining potentially drought-tolerant mutants [9, 27]. In this study, leaflets from fast neutron–irradiated peanut seeds were cultured on somatic embryo induction medium and then on somatic embryo germination medium for mutant screening. Our results indicated that the percentage of both somatic embryos and regenerated plantlets decreased as the radiation dosage increased. The regenerated plantlets were grafted and transplanted into the field. The offspring (the M_2_ generation) of most of the regenerated plants exhibited variations and trait segregation in plant height, plant architecture, branch number, pod shape and pod number per plant.

To determine whether the altered plant traits might be associated with genomic modifications, 19 M_3_ individuals from different regenerated plants were used for the SSR analyses. The specific bands were compared between mutants and wild-type plants (Luhua 11). The SSR analyses indicated that the regenerated plants were genetically different from the wild-type plants. Additional research is needed to determine whether and how the SSR markers were associated with the specific mutant traits.

Although many mutants obtained in this study showed significant variation in growth and pod traits, determining how they differ in agronomic traits, seed quality, and resistance to stresses requires additional research. Our results demonstrate that fast neutron irradiation combined with *in vitro* culture is a promising way to create new peanut mutants that might be useful for breeding elite cultivars.

## FUNDING

This work was supported by the National High Technology Research and Development Program (863 Program) of P. R. China (2012AA101202), the Generation of Mutant Pool of Peanut, Gene mining, Germplasm Innovation and Utilization Project of Shandong, the Natural Science Foundation of P. R. China (31101178), and the Natural Science Foundation of Shandong (ZR2012CM014 and ZR2011CQ026). Funding to pay the Open Access publication charges for this article was provided by Special Fund for Agro-Scientific Research in the Public Interest (201103007).
